# Living Alone, Home Health Visits, and Fall Risk Among Older Adults in New England

**DOI:** 10.1093/geroni/igaf122.2833

**Published:** 2025-12-31

**Authors:** Qinglin Gao, Nachalie Rodriguez Cruz, Calvin Tran, Mengshi Liu, Qian Song, Elizabeth Dugan

**Affiliations:** University of Massachusetts Boston, Boston, Massachusetts, United States; University of Massachusetts Boston, Boston, Massachusetts, United States; University of Massachusetts Boston, Boston, Massachusetts, United States; University of Massachusetts Boston, Boston, Massachusetts, United States; University of Massachusetts Boston, Boston, Massachusetts, United States; University of Massachusetts Boston, Boston, Massachusetts, United States

## Abstract

Falls are a leading cause of injury and hospitalization among older adults, leading to loss of independence, increased healthcare costs, and reduced quality of life. While previous research has examined individual health and physical risk factors, studies on community-level fall rates are limited. Older adults who live alone may face delayed assistance, limited social support, and reduced access to in-home care, increasing fall risk and post-fall complications. This study compares community rates of falls, fall-related injuries, hip fractures, home health visits, and living alone in Massachusetts, Rhode Island, and Connecticut. Data were from the 2025 Healthy Aging Data Reports. Indicators were calculated using data from the Centers for Medicare & Medicaid Services Master Beneficiary Summary File (2020-2021), the American Community Survey (2016-2022), and the Behavioral Risk Factor Surveillance System (2014-2020). Fall rates were similar across states (CT: 26.3%, RI: 26.01%, MA: 26.58%), as were fall-related injuries (CT: 9.8%, RI: 10.03%, MA: 10.06%). Hip fracture rates were highest in Warren, RI (4.07%), Salisbury, CT (5.08%), and Canton, MA (4.52%). Rhode Island had the highest percentage of older adults living alone (29.27%), with Hartford, CT (41.27%) the highest statewide. Home health visits varied, with New Haven, CT (5.39 visits) the highest and Sharon, CT (1.12 visits) the lowest. Findings indicate regional differences in fall-related indicators. Areas with more older adults living alone and fewer home health visits may require targeted support. Further research is needed to explore these trends and inform potential strategies for fall prevention and aging in place support.

